# Structural insight into how WDR4 promotes the tRNA N7-methylguanosine methyltransferase activity of METTL1

**DOI:** 10.1038/s41421-023-00562-y

**Published:** 2023-06-27

**Authors:** Xiaohuan Jin, Zeyuan Guan, Na Hu, Chunjie He, Ping Yin, Zhou Gong, Delin Zhang

**Affiliations:** 1 National Key Laboratory of Crop Genetic Improvement, Hubei Hongshan Laboratory, Huazhong Agricultural University, Wuhan, Hubei China; 2 State Key Laboratory of Magnetic Resonance and Atomic Molecular Physics, Innovation Academy for Precision Measurement Science and Technology Chinese Academy of Sciences, Wuhan, Hubei China

**Keywords:** X-ray crystallography, Epigenetics

Dear Editor,

N7-methylguanosine (m^7^G) at position 46 of the tRNA variable loop is among the most prevalent posttranscriptional tRNA modifications in prokaryotes and eukaryotes, and plays crucial roles in the stability and function of tRNAs^1–3^. In mammals, tRNA m^7^G46 modifications are installed by methyltransferase-like 1 (METTL1) and its cofactor WD repeat domain 4 (WDR4)^4^. WDR4 is indispensable for maintaining normal METTL1 protein levels and the function of METTL1–WDR4 complexes, as depletion of WDR4 decreases METTL1 expression and tRNA m^7^G modification levels^5,6^. Recent studies have revealed that METTL1 or WDR4 deficiency abolishes m^7^G tRNA modification and results in a variety of disorders, including impaired embryonic stem cell self-renewal and differentiation^7^, microcephalic primordial dwarfism and Galloway-Mowat syndrome^8–10^. Additionally, METTL1 and WDR4 are upregulated in a variety of cancer cells and regulate the translation of oncogenes and cell-cycle related mRNAs in an m^7^G tRNA-decoded codon-dependent manner to promote tumor progression^5,6,11,12^. However, the mechanism by which WDR4 regulates the function of METTL1 remains elusive, which limits drug development for m^7^G-related cancers and other diseases.

In this study, we aimed to resolve the structure of the METTL1–WDR4 complex to reveal how WDR4 regulates the methyltransferase activity of METTL1. Solitary METTL1 exhibited good behavior in gel filtration (Supplementary Fig. S1a), indicating its proper folding. The purified protein showed weak methyltransferase activity on transcribed human tRNA^Phe^ as determined by the MTase-Glo assay, while the proposed SAM-binding mutant METTL1 (E107A) exhibited barely detectable activity (Fig. 1a and Supplementary Fig. S1b, c). Interestingly, our results are inconsistent with those reported by the recent work of Ruiz-Arroyo et al.^13^. We further verified our findings with Liquid Chromatography-Mass Spectrometry (LC-MS, Supplementary Fig. S1d, e). The production of m^7^G could not be detected in the reaction using modified substrates, indicating G46 as the site catalyzed by METTL1. Interaction between METTL1 and WDR4 was revealed by the co-elution of WDR4 with METTL1 in gel filtration (Supplementary Fig. S1a). The addition of WDR4 significantly increased the methyltransferase activity of METTL1 (Fig. 1a). The mutant METTL1 (E107A)–WDR4 exhibited notable impairment of activity and METTL1–WDR4 showed no detectable activity on the modified substrate tRNA (Fig. 1a and Supplementary Fig. S1e).

**Fig. 1:**
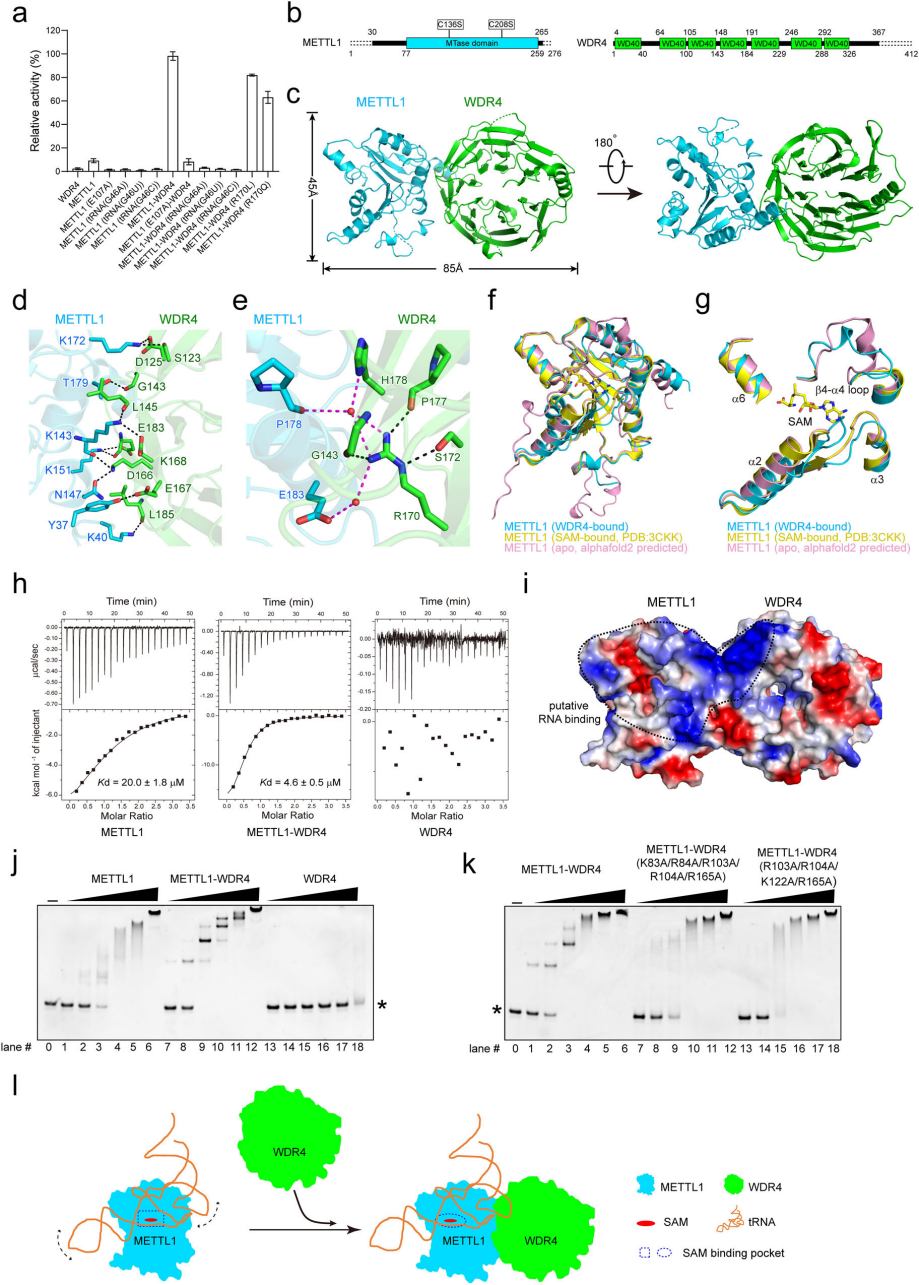
WDR4 promotes the tRNA N7-methylguanosine methyltransferase activity of METTL1. **a** Relative methyltransferase activity of METTL1, WDR4 and the METTL1–WDR4 complex. **b** Schematic domain structures of METTL1 and WDR4. The truncated regions for crystallization of METTL1 and WDR4 are represented by dashed lines. **c** Overall structure of the METTL1–WDR4 heterodimer. Dashed lines indicate residues with missing electron density. **d** Close-up view of the interface of METTL1 and WDR4. Residues forming the interface of METTL1 and WDR4 are shown as cyan and green sticks, respectively. Dashed lines represent hydrogen bonds. **e** Schematic representation of the interactions between R170 on WDR4 and other residues on METTL1 (cyan) and WDR4 (green). Water is shown as a red ball. **f** Superimposition of the WDR4-bound (cyan), SAM-bound (yellow, PDB: 3CKK) and apo (pink, alphafold2 predicted) structures of METTL1. SAM is shown as yellow sticks. **g** Close-up view of the conformational change regions shown in **f**. **h** ITC measurement of the binding affinity between SAM and METTL1, the METTL1–WDR4 complex and WDR4. **i** Electrostatic surface of the METTL1–WDR4 complex with the potential RNA-binding surface marked by a dashed circle. Blue, white, and red represent positive, neutral, and negative surfaces, respectively. **j**, **k** EMSA analysis of the interaction between tRNA^phe^ and proteins. *indicates free tRNA^phe^. **l** The proposed mechanism by which WDR4 promotes the methyltransferase activity of METTL1. Dashed arrows indicate the dynamics of the tRNA.

To determine the structure of the METTL1–WDR4 complex, we expressed and purified full-length METTL1–WDR4 in *Escherichia coli* (Supplementary Fig. S2a). However, we failed to resolve either the structure of integral METTL1–WDR4 or its complex with SAM or substrate tRNA^Phe^. After a series of screening and optimization, we successfully determined the crystal structure of the METTL1^30-265 (C136S, C208S)^–WDR4^1–367^ boundary at a global resolution of 1.8 Å with an R_work_ of 0.1931 and an R_free_ of 0.2263 (Fig. 1b and Supplementary Table S1). The METTL1–WDR4 complex consists of one METTL1 molecule and one WDR4 molecule and exhibits a symbol “∞” shape with a width of ~45 Å and a length of ~85 Å (Fig. 1c). Static light scattering (SLS) analyses revealed that the molecular mass of the METTL1–WDR4 complex in solution is ~84.6 kDa (Supplementary Fig. S2b), which is consistent with the crystal structural information. Dali search results suggest that the METTL1–WDR4 complex mostly resembles the yeast tRNA m^7^G methyltransferase complex Trm8–Trm82 (PDB: 2VDU). The root-mean-square deviation (RMSD) for METTL1 and Trm8 is 0.636 Å over 137 Cα atoms, while the RMSD for WDR4 and Trm82 is 3.038 Å over 189 Cα atoms (Supplementary Fig. S3a).

The METTL1–WDR4 structure resolved in this study is overall superimposed with that reported by two newly published studies^13,14^, though with some regional differences. For METTL1, the major difference was the β2-α2 loop, which formed an extended loop in this study (Supplementary Fig. S3b, c). The catalytic loop (β4-α4 loop) is divergent, which is partially invisible and “closed” in Li’s study^14^ while visible and “open” in Ruiz-Arroyo’s^13^ and our studies (Supplementary Fig. S3b, c). These differences may result from the flexibility of the loops.

The METTL1 N-terminal region is highly flexible, in which residues 37–57 exist mainly as a long loop. METTL1 residues 77–259 adopt a typical SAM-dependent Class I methyltransferase fold, which consists of seven conserved stranded β sheets (β6 ↑ , β7 ↓ , β5 ↓ , β4 ↓ , β1 ↓ , β2 ↓ , β3 ↓ ) flanked by six α-helices (α1, α2, and α6 on one side, and α3, α4, and α5 on the other side) and five short helices (Supplementary Fig. S4a). WDR4 in the METTL1–WDR4 complex displays an overall β-propeller architecture, which consists of seven blades and blade 7 is followed by a long α-helix (residues 331–349) embedded in the interface between blade 1 and blade 7 (Supplementary Fig. S4b). The η3, α3, and α4 regions of METTL1 interact with WDR4 blade 3- and blade 4-related strands and loops (Supplementary Fig. S4c). The interface of METTL1 and WDR4 is composed of hydrogen bonds and hydrophobic and electrostatic interactions, with a buried area of ~1004.4 Å^2^ (Fig. 1d and Supplementary Fig. S5a, b). Residues that participate in hydrogen bonds and salt bridge interactions are highly conserved among different species (Supplementary Figs. S6, S7). The mutant METTL1 (K40D/K143D/K151D/K172D) lost its capacity to co-elute with wild-type WDR4 in affinity chromatography assay (Supplementary Fig. S8a) or gel filtration (Supplementary Fig. S8b).

It has been reported that the R170L or R170Q mutation in WDR4 could lead to impaired tRNA m^7^G46 methylation and result in a distinct form of microcephalic primordial dwarfism^8,9^. Consistent with previous studies, the methyltransferase activity of the WDR4 (R170L) and WDR4 (R170Q) mutants was apparently decreased (Fig. 1b). We further inspected the R170-interacting residues within the METTL1–WDR4 complex and found that R170 interacted extensively with other residues within the METTL1–WDR4 complex (Fig. 1e and Supplementary Fig. S5c). In yeast, the corresponding residue K223 (R170) of Trm82 (homolog of WDR4) forms a salt bridge with residue E204 (E183) of Trm8 (homolog of METTL1)^15^ (Supplementary Fig. S5d). The WDR4 R170-related mutations may have affected the stability or folding of the METTL1–WDR4 complex and thus influenced its methyltransferase activity.

To investigate whether WDR4 binding could induce a conformational change in METTL1, we compared the structure of METTL1 in the WDR4-bound state with that in the apo state (Alphafold2 predicted) and SAM-bound state (PDB ID: 3CKK). The structural comparison showed that the conformation of METTL1 in the WDR4-bound state exhibits overall similarity to that in the apo or SAM-bound state. However, their active site, which harbors the SAM-binding pocket showed some major conformational differences (Fig. 1f, g). First, METTL1 residues 107–112 (EIRVKV), which are important for SAM binding and recognition, formed an extended loop in the WDR4-bound state, instead of a short loop in apo or SAM-bound METTL1 (Fig. 1g and Supplementary Fig. S9a). For residue E107, an ~2 Å shift of Cα and a change in side chain orientation in the WDR4-bound structure were observed compared to apo METTL1. The Cα of I108 shifted ~5 Å inward from the SAM-binding pocket in the WDR4-bound state, and this side chain conformation clashes with the adenine base of SAM in METTL1-SAM. R109 also displayed a conformational change in a manner similar to I108 (Supplementary Fig. S9a). Second, apart from the METTL1 EIRVKV loop, the loop containing N140 and A141 located between β3 and α3 exhibited a shift away from the SAM adenine base, and METTL1 α6 containing T238 and E240 showed a subtle shift away from the carboxyl group of the methionine moiety in the WDR4-bound state (Supplementary Fig. S9b, c). Finally, a distinct conformational difference was observed in the region of the β4-α4 loop (residues 161–175) between the WDR4-bound and apo states of METTL1, which was invisible in the SAM-bound state (Fig. 1g and Supplementary Fig. S9d–h).

The conformational change of METTL1 induced by WDR4 binding, especially in the vicinity of the SAM-binding pocket, indicated that WDR4 may affect the SAM-binding ability of METTL1. Our isothermal titration calorimetry (ITC) results revealed that WDR4 notably increased the binding affinity between METTL1 and SAM. The *K*_d_ values for SAM binding with METTL1 alone and with the METTL1–WDR4 complex are ~20 μM and ~4 μM, respectively (Fig. 1h). The enhanced SAM-binding affinity of METTL1 induced by WDR4 could specifically contribute to the methyltransferase activity of the METTL1–WDR4 complex. These results may also explain why individual METTL1 has detectable enzymatic activity in this study, while it was not found to be active in the report of Ruiz-Arroyo et al. The METTL1/SAM molar ratio used in this study (5/0.5 μM) was much higher than that of Ruiz-Arroyo et al. (230/100 nM), making it more likely to detect the relatively weak activity of METTL1. The individual mutations in the SAM-binding site and in the β4-α4 loop significantly impaired the methyltransferase activity of the METTL1–WDR4 complex (Supplementary Fig. S9i).

We found a large patch of positively charged residues spanning from the entrance of the SAM-binding pocket on METTL1 to WDR4 blade 3 and blade 4 (Fig. 1i and Supplementary Fig. S10a), suggesting that WDR4 could act as a scaffold to facilitate substrate tRNA binding. EMSA results showed that METTL1 alone binds with tRNA^Phe^, with heterogeneous METTL1–tRNA^Phe^ bands when the protein concentration is low (Fig. 1j). The METTL1–WDR4 complex exhibited not only a stronger affinity for substrate tRNA but also distinct bands compared to solitary METTL1 (Fig. 1j), indicating that WDR4 contributes to substrate tRNA binding of the METTL1–WDR4 complex. Mutation on residues of WDR4 that interact with tRNA resulted in altered patterns of METTL1–WDR4 binding with tRNA (Fig. 1k) and impaired methyltransferase activity of the METTL1–WDR4 complex (Supplementary Fig. S10b), while the SAM-binding affinity of the mutated METTL1–WDR4 complex was not affected (Supplementary Fig. S10c). These results confirmed that WDR4 directly participates in substrate binding and facilitates the docking of tRNA to the proper position of the METTL1–WDR4 complex for efficient catalysis.

In summary, our structural and biochemical analyses revealed that WDR4 promotes the methyltransferase activity of METTL1 by simultaneously enhancing SAM recognition and facilitating substrate RNA binding (Fig. 1l). Our results provide important insights into the mechanisms by which WDR4 contributes to the methyltransferase activity of the METTL1–WDR4 complex, and the development of drugs targeting tRNA m^7^G-related diseases.

## Supplementary information

Supplementary Information

## Data Availability

Protein coordinates are deposited at the Protein Data Bank (8H0N).
