# Supplementary material for: Structural insight into how WDR4 promotes the tRNA N7-methylguanosine methyltransferase activity of METTL1
Source: Cell Discov. 2023 Jun 27;9:65. doi: 10.1038/s41421-023-00562-y (PMC10300002; doi:10.1038/s41421-023-00562-y)
Supplement: Supplementary file 1 — Supplementary Information [file 41421_2023_562_MOESM1_ESM.pdf]

Supplementary Materials for

Structural insight into how WDR4 promotes the tRNA N7-methylguanosine methyltransferase activity of METTL1

MATERIAL AND METHODS

Molecular cloning, protein expression and purification

Full-length human METTL1 (Q9UBP6) and WDR4 (P57081) genes were codon-optimized and synthesized by General Bio-systems Company. For crystallization, the truncated METTL1 (M30-Q265) harboring mutations (C136S, C208S) and truncated WDR4 (M1-N367) were sub-cloned into pET21b vector with a C- terminal 8 × His tag and pBB75 vector without any tag, respectively. All site-directed mutagenesis of METTL1 and WDR4 were carried out using the fusion PCR method. In vitro methylation experiments, for the METTL1-WDR4 complex, METTL1 and WDR4 were sub-cloned into pBB75 vector without any tag and pET21b vector with a C-terminal 8 × His tag, respectively. For solitary METTL1 or WDR4, it was sub-cloned to the pET21b vector with a C- terminal 8 × His tag.

Proteins were overexpressed in *E. coli* BL21 (DE3) and induced with 0.2 mM isopropyl-β-D-thiogalactopyranoside (IPTG) for 16 h at 16°C. The bacterial cells were collected and homogenized (JNBIO, China) in buffer A (25 mM Tris-HCl, pH 8.0, 150 mM NaCl) with 1 mM PMSF. After centrifuged at 14,000 rpm for 1 h at 4°C, the supernatant was loaded onto a Ni-NTA affinity column (Qiagen, Germany), which was pre-equilibrated with buffer A. The protein was washed with buffer B (25 mM Tris-HCl, pH 8.0, 150 mM NaCl, 15 mM imidazole), and eluted with buffer C (25 mM Tris-HCl, pH 8.0, 250 mM imidazole). The eluted proteins were further purified via anion exchange chromatography (Source 15 Q10/100, GE healthcare) with a linear NaCl gradient. The elution peak was concentrated to 1 ml and subjected to gel filtration chromatography (Superdex™ 200 10/300, GE Healthcare) equilibrated with 25 mM Tris-HCl, pH 8.0, 150 mM NaCl and 5 mM 1,4-dithiothreitlo (DTT).

Crystallization

Initial crystallization screening for METTL1-WDR4 complex were performed manually using the hanging-drop vapor-diffusion method at 18°C, with the Crystal, PEG/Ion, ProPlex, SaltRxl, Index, and Wizard Classic screens (Hampton Research, USA). Crystals of $METTL1^{30\text{-}265\ (C136S,\ C208S)}$-$WDR^{41\text{-}367}$ complex were obtained from the drops consisting of 1.4 µl of protein solution with equal volume of reservoir solution containing 0.1M Bis-Tris, (pH 5.0-6.0), 0.2 M $LiSO_4$, and 15-25% (w/v) PEG3350. The crystals were flash-cooled in cryoprotectant made of the reservoir solution supplemented with 25% ethylene glycol (EG).

Data collection and structure determination

X-ray diffraction datasets were collected at the Shanghai Synchrotron Radiation Facility (SSRF) on beamline BL17 U and processed using the HKL3000 program suit and XDS packages[1]. METTL1-WDR4 complex structure was solved by molecular

replacement with PHASER using the structure of yeast Trm8-Trm82 (PDB: 2VDU) as the initial searching model[2]. Crystal structures were built using COOT[3] and refined using the PHENIX program[4]. Data collection and structure refinement statistics are summarized in Table S1. All structural figures were generated by PyMOL (http://www.pymol.org/).

In vitro transcription and purification of tRNA^{phe}

The sequences of the gene encoding human phenylalanine tRNA (tRNA^{phe}: 5′-pppGCCGAAAUAGCUCAGUUGGGAGAGCGUUAGACUGAAGAUCUAAAG**G**UCCCUGGUUCGAUCCCGGGUUUCGGCACCA-3′; the bold underlined **G** indicates the G46 site subjected to m^{7}G modification) was obtained from the tRNAdb (Transfer RNA database). tRNA^{phe} and tRNA^{phe} mutants (in which the G46 of the substrate tRNA^{Phe} is substituted with A46, U46 or C46) were synthesized by in vitro T7 RNA polymerase run-off transcription as described previously[5]. The templates for the in vitro transcription of all tRNAs were generated by PCR amplification and phenol-extracted. The transcription reaction was performed at 37°C for 8 h in a mixture containing 100 mM Tris-HCl, pH 8.0, 20 mM $MgCl_2$, 2 mM spermidine, 4 mM DTT, 0.01% (v/v) Triton X-100, 4 mM nucleotides (ATP, GTP, CTP and UTP), PCR product of the templates (0.1 mg ml^{-1}) and 1 µM T7 RNA polymerase. The transcribed tRNA product was purified by electrophoresis in a 12% denatured Urea-PAGE gel, extracted with 0.5 M NH_4AC by passive diffusion and concentrated by ethanol precipitation. The pellet was washed and redissolved in 5 mM $MgCl_2$. Finally, the tRNA was refolded by rapid heating at 95°C for 3 min and slow cooling to 25°C.

In vitro methylation activity assays (MTase-Glo assay)

The methylation activity assay was performed using the MTase-Glo assay (Promega)[6]. In brief, tRNA^{phe} (1 µM) were incubated with purified proteins (0.5 µM) in the presence of SAM (5 µM) for one hour at 37°C in a 10 µl reaction buffer (contained 85 mM Tris-HCl, pH 8.0, 1.4 mM DTT, 0.07 mM EDTA, 1mM spermidine and 1 × MTase-Glo Reagent). Then, 10 µl MTase-Glo Detection solution was added and mixed well before incubation for another 30 min at room temperature and recording luminescence by a TECAN infinite M200 (TECAN). The value of reaction in absence of the native tRNA^{Phe} was subtracted as background for measurement of the mutated enzymes. The value of reaction without SAM was subtracted as background for measurement of the modified substrates including tRNA^{Phe} (G46A), tRNA^{Phe} (G46U) and tRNA^{Phe} (G46C).

In vitro methylation activity assays (LC-MS)

tRNA^{Phe} (1 µM) were incubated with purified proteins (0.5 µM) in the presence of SAM (5 µM) for one hour at 37°C in a 10 µl reaction buffer (contained 85 mM Tris-HCl, pH 8.0, 1.4 mM DTT, 0.07 mM EDTA, 1mM spermidine). After reaction, the sample was diluted with additional 200 µl ddH_2O, and quenched with 200 µl of 1:1 (v/v) Tris-phenol (pH 8.0):chloroform. The supernatant was transferred to a new tube and concentrated by ethanol precipitation. The precipitated RNA was dissolved in 15 µl ddH_2O, and digested with P1 nuclease (N8630, Sigma-Aldrich) and calf intestine alkaline

phosphatase (18009-027, Invitrogen) in 25 mM NH_4HCO_3 (pH 8.0) at 37°C for 3 h[7]. After the incubation, the sample was quenched with 1:1 (v/v) Tris-phenol (pH 8.0):chloroform and injected into TRIPLE QUAD™ 5500 mass spectrometer coupled with LC-20AD HPLC system for nucleoside separation and detection. The respective mass transitions used for quantification of m^7G and G were *m/z* 298.300/166.100 and 284.300/152.100, respectively. The identity of m^7G peak was confirmed with external standard (20244-86-4, Santa Cruz Biotechnology, Inc. USA).

RNA electrophoretic mobility shift assay (EMSA)

Purified protein (final concentrations: 0, 0.125, 0.25, 0.5, 0.75, 1, 1.5, 2 μM) and 0.25 μM $tRNA^{phe}$ were incubated on ice for 30 min in 10 μl buffer containing 25 mM Tris-HCl, pH 8.0, 1 mM DTT, 5 mM EDTA, 0.2 mg/ml Heparin and 10% glycerol. Samples were loaded onto a 8% native-PAGE gel, the electrophoresis was performed at 4°C for 3 h at 15 v/cm after pre-running the gel for 30 min, with 0.5% Tris-glycine (TG) buffer as the running buffer, and the signals were stained with Gel-Red (Sangon Biotech, A616697).

Isothermal titration calorimetry (ITC) assays.

ITC experiments for the binding of SAM to the METTL1-WDR4 complex or individual METTL1 and WDR4 were performed at 25°C using Auto-iTC200 titration calorimetry (Malvern). SAM (200 μM) was dissolved in a buffer containing 25 mM Tris-HCl, pH 8.0, and 150 mM NaCl (150 μl) and titrated against 30 μM wild-type or mutant METTL1-WDR4 complex (350 μl) in the same buffer. One injection with 0.4 μl and followed by 19 injections of 2 μl were performed with an equilibration break of 150 s between subsequent injections. The heat of dilution values for SAM was measured by injecting SAM into the buffer alone. The values were subtracted from the experimental curves before data analysis. The stirring rate was 750 r.p.m. The MicroCal ORIGIN software supplied with the instrument was used to determine the site-binding model that produced a good fit (low×2 value) for the resulting data.

Additional References

1 Otwinowski Z. & Minor W. Processing of X-ray diffraction data collected in. *Methods Enzymol* **276**, 307-326 (1997).

2 McCoy, A. J. *et al.* Phaser crystallographic software. *J. Appl. Crystallogr.* **40**, 658-674 (2007).

3 Emsley, P. & Cowtan, K. Coot: model-building tools for molecular graphics. *Acta Crystallogr. D Biol. Crystallogr.* **60**, 2126-2132 (2004).

4 Adams P.D. *et al.* PHENIX: building new software for automated crystallographic structure determination. *Acta Crystallogr. D Biol. Crystallogr.* **58**, 1948-1954 (2002).

5 Nilsen, T. W., Rio, D. C. & Ares, M., Jr. High-yield synthesis of RNA using T7 RNA polymerase and plasmid DNA or oligonucleotide templates. *Cold Spring*

Harb. Protoc. **2013** (2013).

6 Hsiao K., Zegzouti H. & S.A., G. Methyltransferase-Glo a universal, bioluminescent and homogenous assay for monitoring all classes of methyltransferases. *Epigenomics* **8**, 321-339 (2016).

7 Xiaohuan Jin, Z. L., Junbao Gao, Rui Zhang, Ting Zheng, Ping Yin, Dongqin Li, Liangcai Peng, Xintao Cao, Yan Qin, Staffan Persson, Bo Zheng, Peng Chen. AtTrm5a catalyses 1-methylguanosine and 1-methylinosine formation on tRNAs and is important for vegetative and reproductive growth in Arabidopsis thaliana. *Nucleic Acids Res.* **47**, 16 (2019).

Supplementary Table S1. Crystal data collection and final refinement parameters.

Wavelength	0.97918
Resolution range	28.9 - 1.8 (1.864 - 1.8)
Space group	P 2 21 21
Unit cell	84.465 87.03 95.915 90 90 90
Total reflections	132060 (13017)
Unique reflections	66112 (6523)
Multiplicity	2.0 (2.0)
Completeness (%)	99.91 (99.98)
Mean I/sigma(I)	15.03 (5.57)
Wilson B-factor	22.66
R-merge	0.02884 (0.1105)
R-meas	0.04079 (0.1563)
R-pim	0.02884 (0.1105)
CC1/2	0.997 (0.976)
CC*	0.999 (0.994)
Reflections used in refinement	66096 (6522)
Reflections used for R-free	3200 (342)
R-work	0.1931 (0.2114)
R-free	0.2263 (0.2545)
CC (work)	0.951 (0.927)
CC (free)	0.930 (0.841)
Number of non-hydrogen atoms	4751
macromolecules	4251
ligands	0
solvent	500
Protein residues	540
RMS(bonds)	0.006
RMS(angles)	0.86
Ramachandran favored (%)	97.17
Ramachandran allowed (%)	2.83
Ramachandran outliers (%)	0
Rotamer outliers (%)	3.72
Clash score	4.62
Average B-factor	34.13
macromolecules	33.71
solvent	37.71
Number of TLS groups	1

*The values in parentheses indicate the highest resolution shell.

$R_{merge}=\Sigma h \Sigma i|Ih,i-Ih|/\Sigma h \Sigma i Ih,i$,, where I_h is the mean intensity of the *i* observations of symmetry-related reflections of *h*.

$R=\Sigma|F_{obs}-F_{calc}|/\Sigma F_{obs}$, where F_{obs} and F_{calc} are observed and calculated protein structure factor from the atomic model (R_{free} was calculated with 5% of the reflections selected).

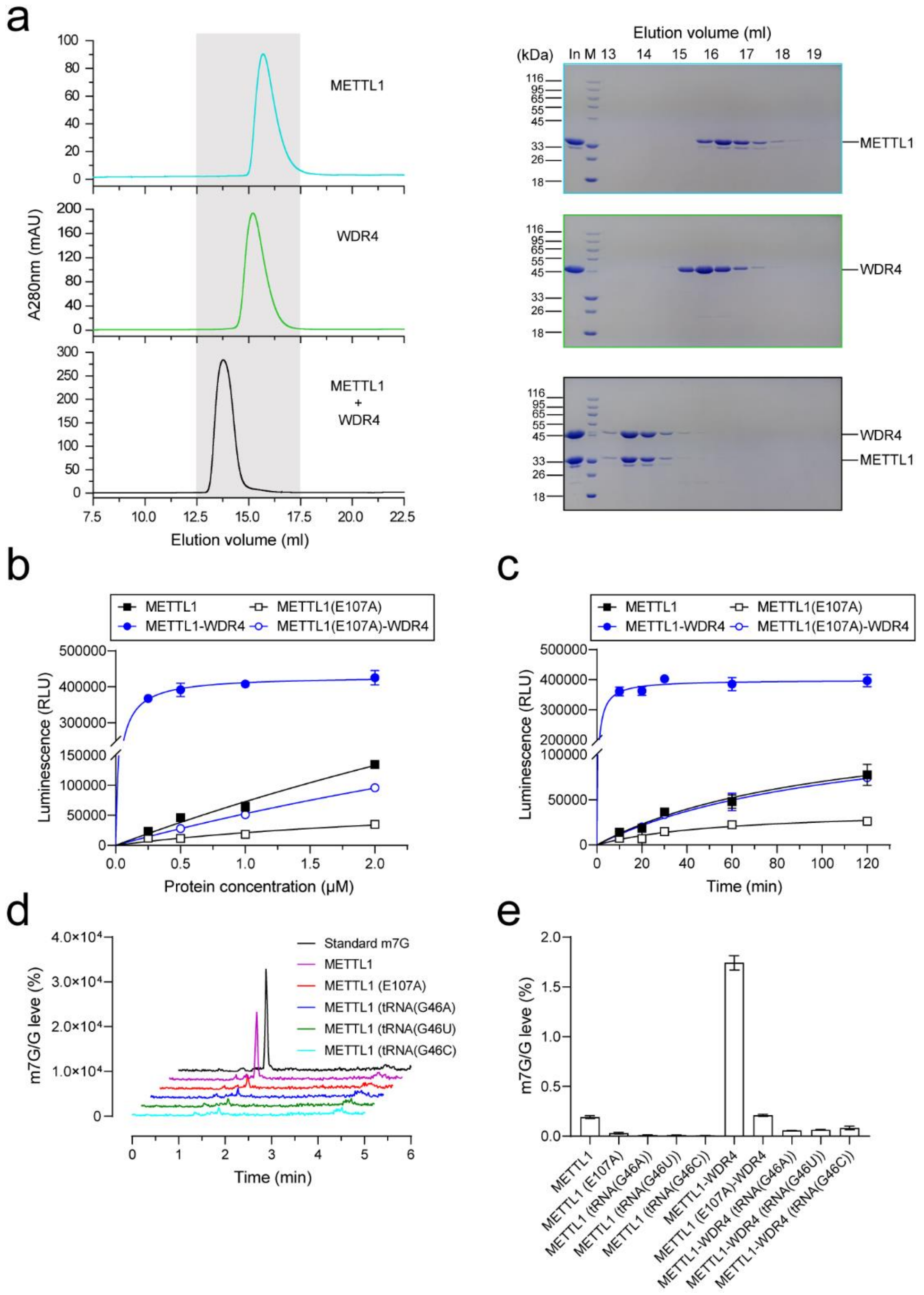

Supplementary Fig. S1 Protein purification and in vitro methylation. **a** Gel filtration chromatography analysis of METTL1, WDR4 and the interaction of METTL1 and WDR4. The left panel lists the profiles of samples applied to gel filtration, and the right panel is the corresponding eluted proteins identified by SDS-PAGE and Coomassie brilliant blue staining. **b** Methyltransferase activity with gradient protein concentration. [tRNA]= 1 μM, reaction for 1 h. **c** Methyltransferase activity with different reaction time. [tRNA]= 1 μM, [protein]= 0.5 μM. **d** Mass spectrometry profiles of m^7G nucleoside generation by METTL1 and the mutants. **e** Quantification of m^7G relative to G nucleoside produced by METTL1 and the METTL1-WDR4 complex.

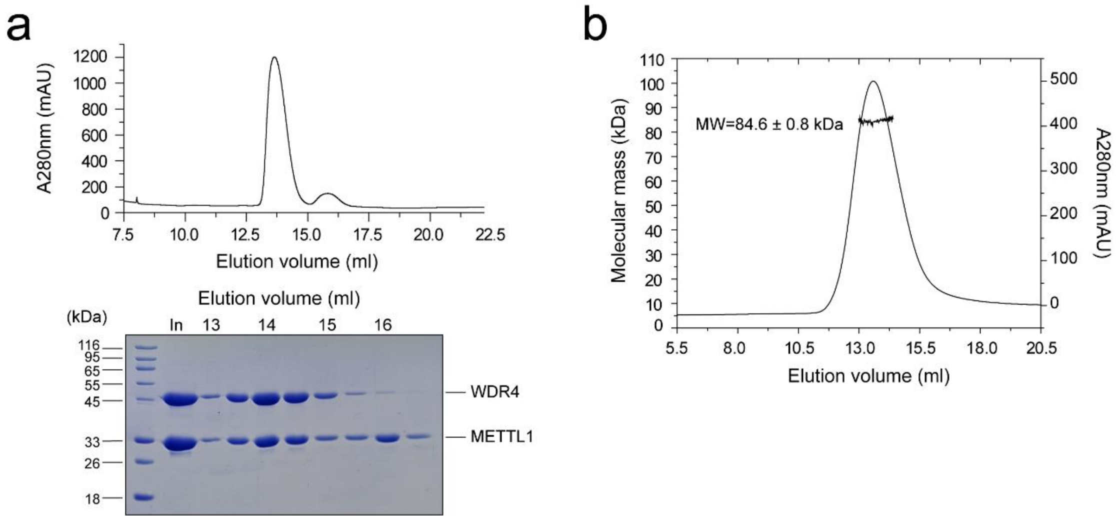

Supplementary Fig. S2 Characterization of the METTL1-WDR4 complex. a Characterization of the purified full-length METTL1-WDR4 complex. The up panel lists the profiles of gel filtration using a superdex^{TM} 200 increase 10/300 GL column; the down panel is the corresponding eluted proteins detected by SDS-PAGE. **b** Static light scattering analyses of the full-length METTL1-WDR4 complex in solution. Run was performed in a superdexTM 200 increase 10/300 GL column. The corresponding METTL1-WDR4 molecular weight is 84.6 ± 0.8 kDa.

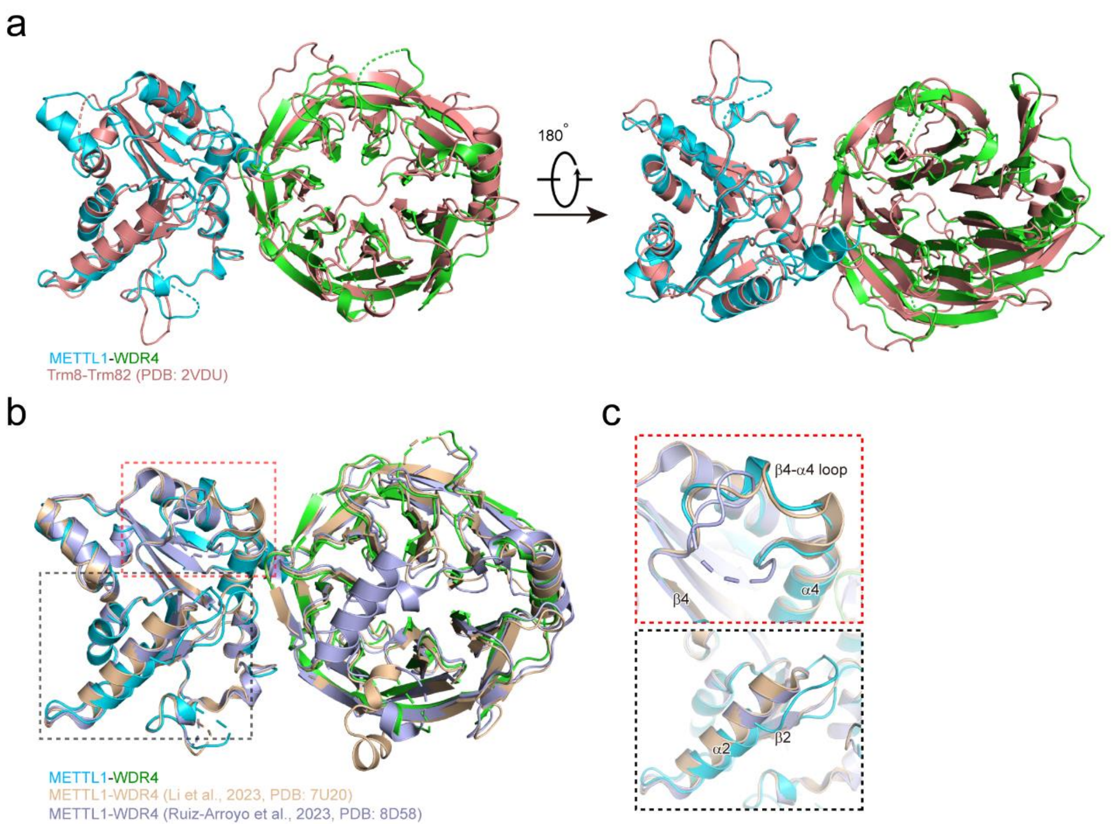

Supplementary Fig. S3 Structure comparisons of METTL1-WDR4 complex. a Structural superposition of human METTL1-WDR4 complex with S.cerevisiae Trm8-Trm82 complex (PDB: 2VDU). **b** Structural superposition of the METTL1-WDR4 complex reported by different studies. **c** Close-up views of β2-α2 loop and β4-α4 loop of METTL1.

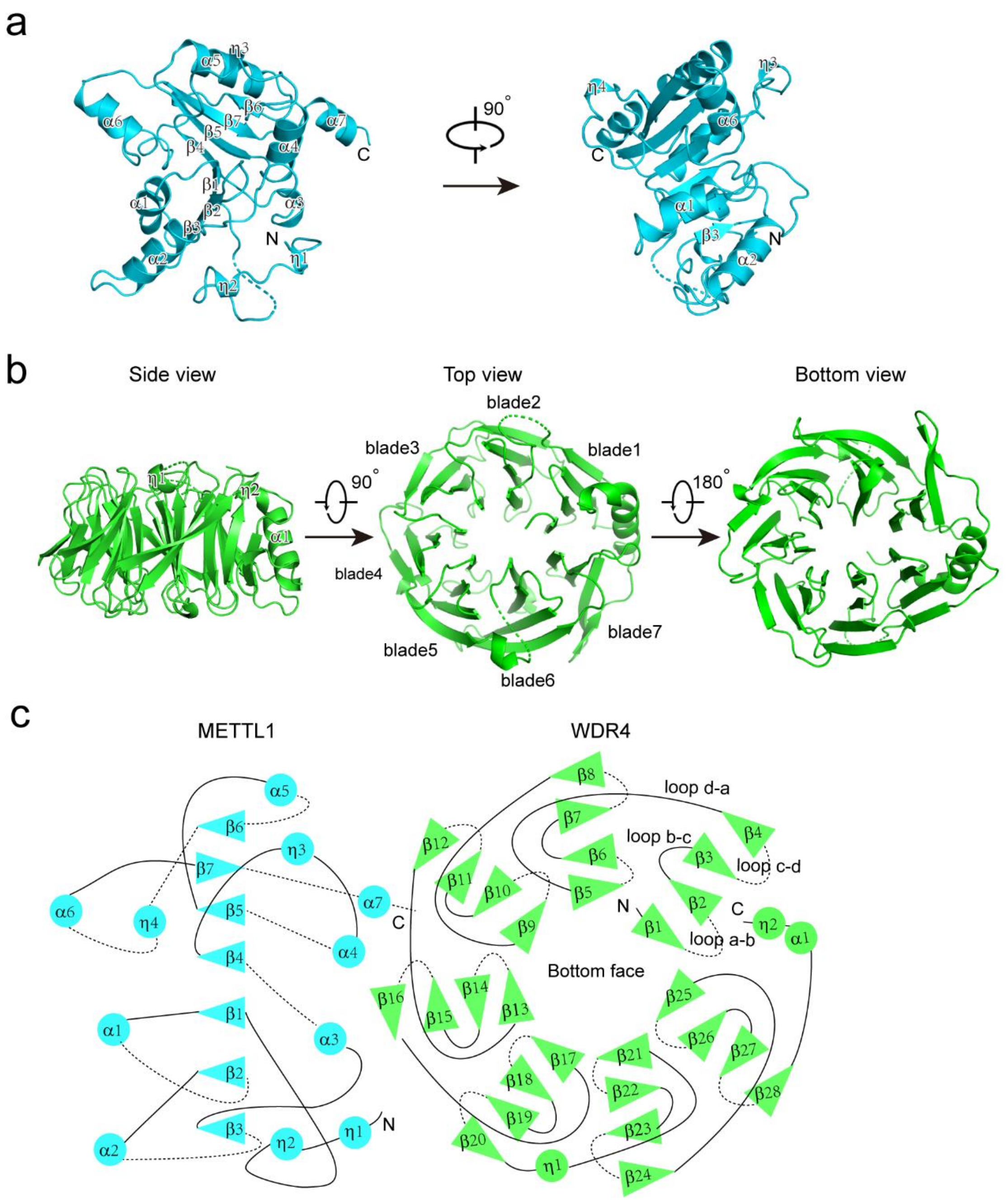

Supplementary Fig. S4 Structure analysis of the METTL1-WDR4 complex. a METTL1 structure in two perpendicular views. Secondary structural elements are labelled. **b** WDR4 structure in different views. Seven blades are labelled. **c** Topological diagram of the METTL1-WDR4 complex secondary structure profiles. Structural elements are numbered by their linear order in the sequence. The loops in the front are indicated by black lines, and loops in the back are indicated by black dashed lines.

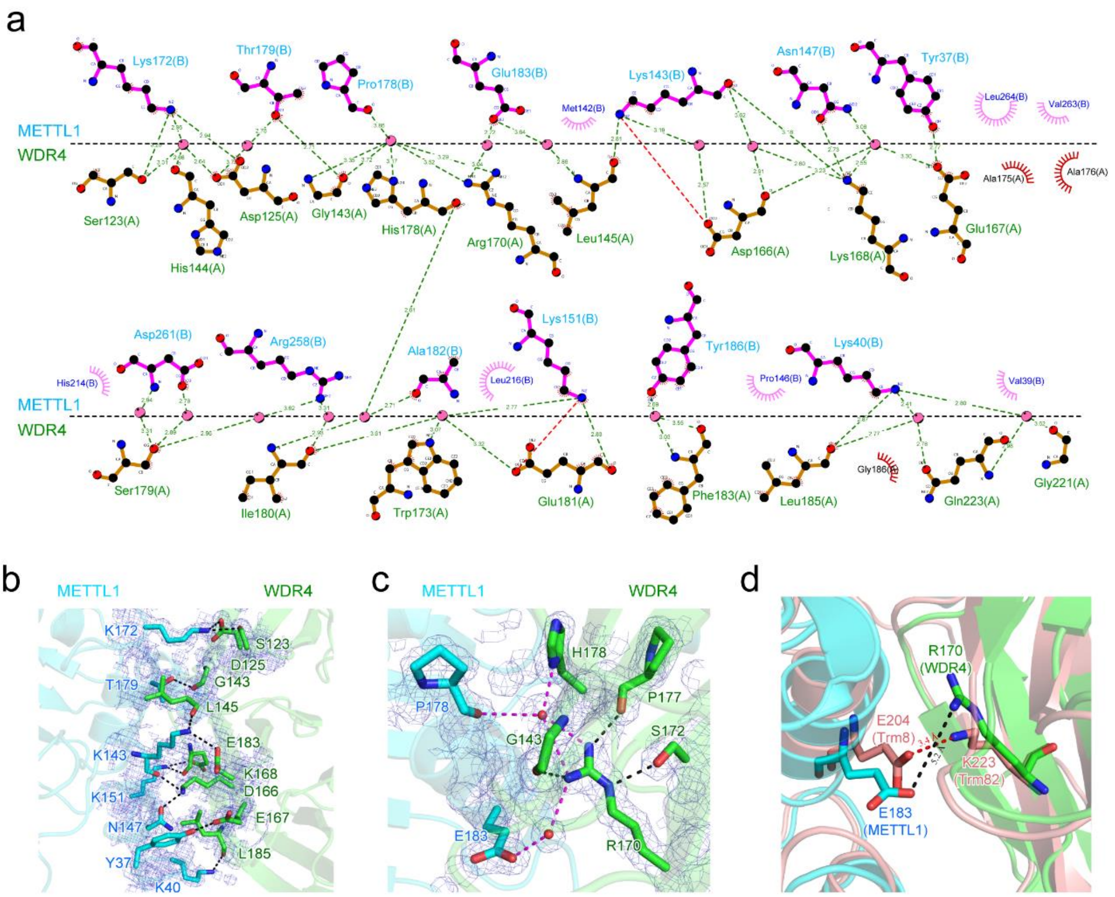

Supplementary Fig. S5 Detailed interactions between METTL1 and WDR4. a Interface analysis using the program of Ligplot+. Hydrogen bonds are shown as green dotted lines, salt bridge are shown as red dotted lines, spoked arcs represent residues making nonbonded contacts. Water molecules are displayed in pink circles on the center line. **b** Interface of METTL1 and WDR4 with real density map shown in mesh. **c** WDR4 R170 interactions with real density map shown in mesh. **d** Superposition of the METTL1-WDR4 complex (METTL1, cyan; WDR4, green) structure and Trm8-Trm82 (PDB: 2VDU, salmon).

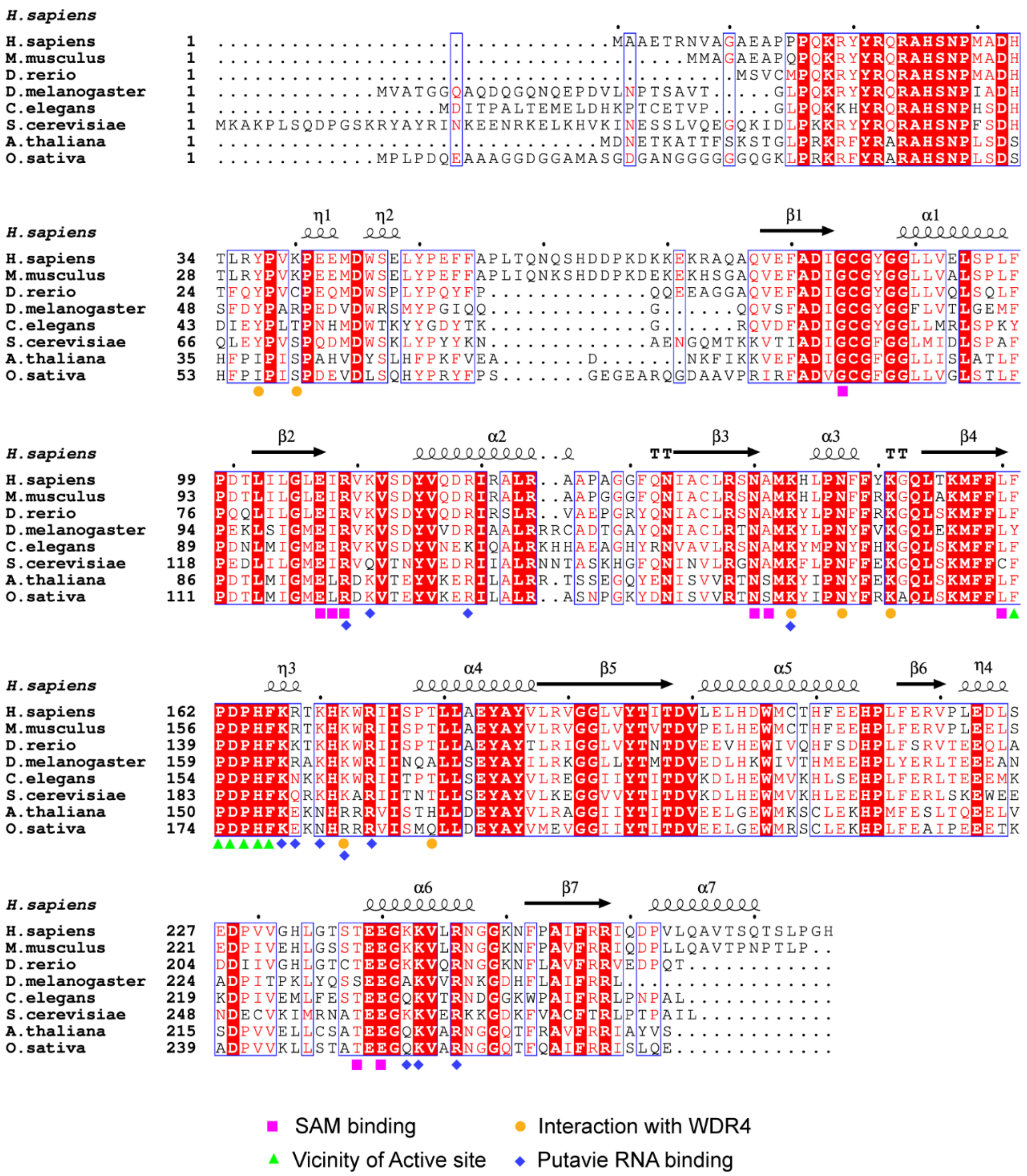

Supplementary Fig. S6 Sequence alignment of METTL1 homologs. The secondary structural elements of human METTL1 are indicated on top. Completely conserved amino acids are colored in red, and partially conserved amino acids are enclosed in blue boxes. SAM binding residues of METTL1 are marked by magenta squares; residues are involved in protein interaction with WDR4 are indicated by orange dots; green triangles indicated residues that vicinity of active site; putative RNA binding residues are highlighted by blue diamonds.

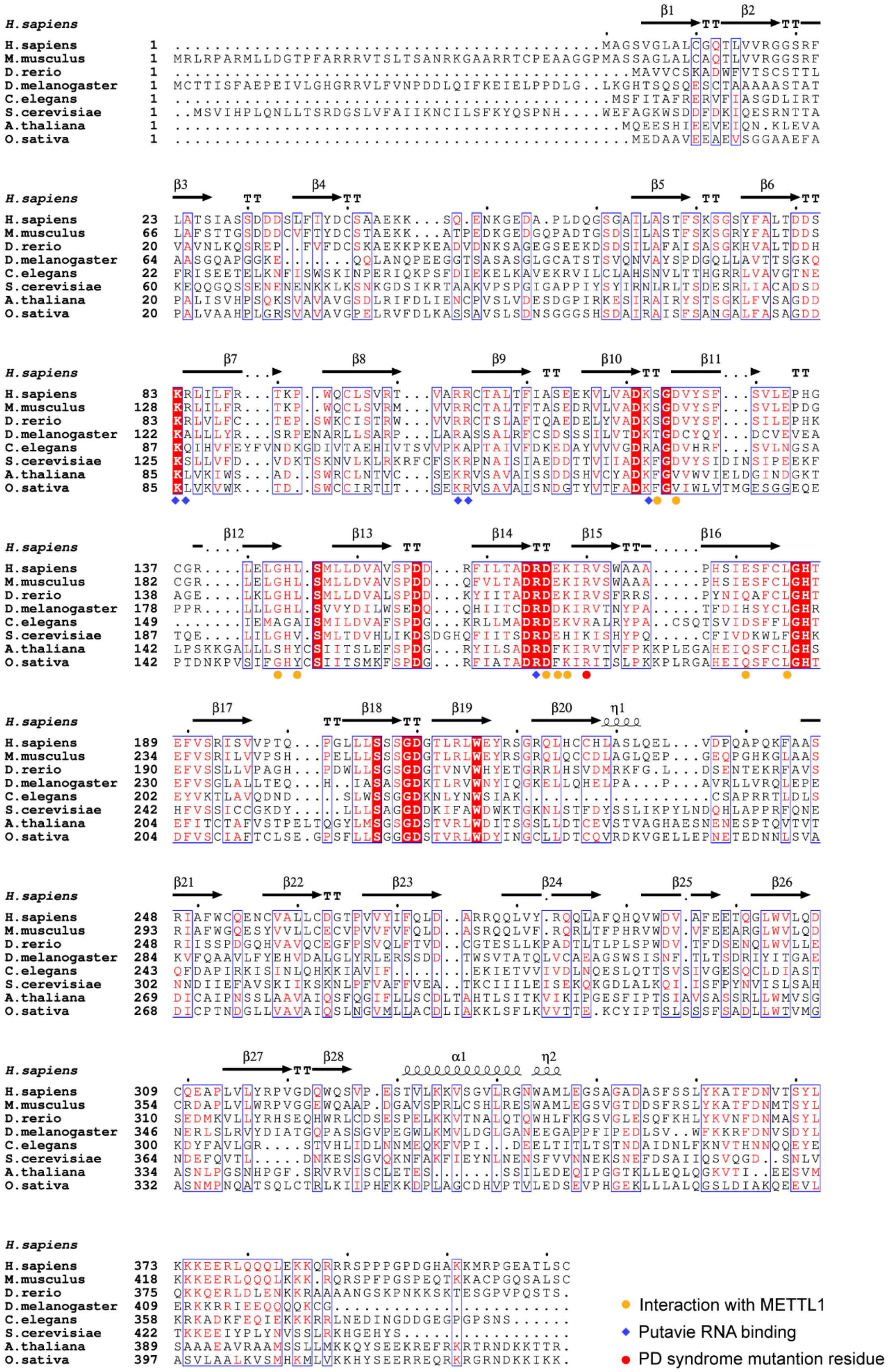

Supplementary Fig. S7 Sequence alignment of WDR4 homologs. The secondary structural elements of human WDR4 are indicated on top. Completely conserved amino

acids are colored in red, and partially conserved amino acids are enclosed in blue boxes. Residues that involved in protein interaction with METTL1 are indicated by orange dots; putative RNA binding residues are highlighted by blue diamonds.

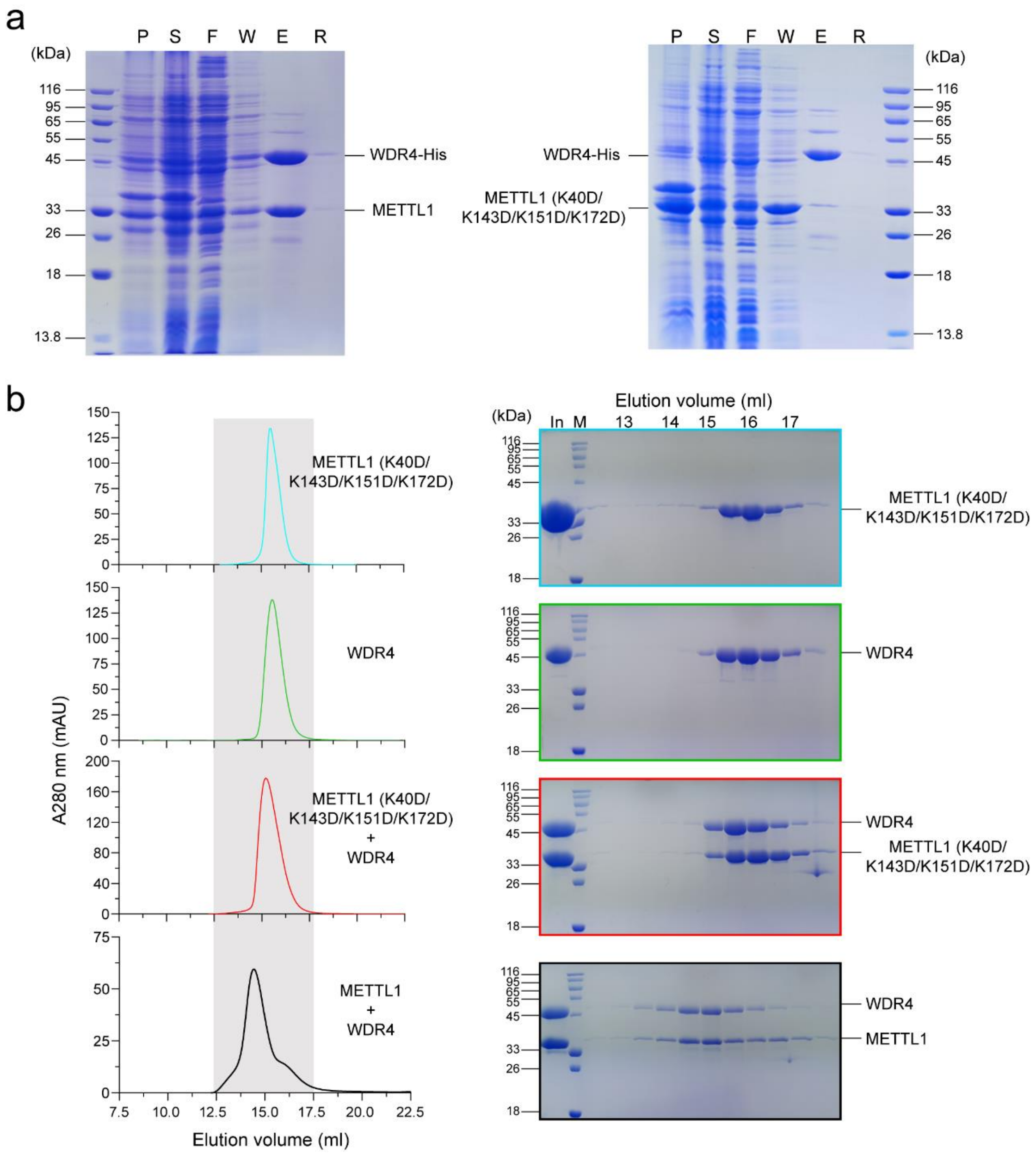

Supplementary Fig. S8 Mutations influence the interaction between METTL1 and WDR4. a Affinity chromatography analysis of the interaction between METTL1 and WDR4 (left panel); METTL1 (K40D/K143D/K151D/K172D) and WDR4 (right panel). P: precipitate, S: supernatant, F: flow through, W: wash, E: elute, R: resin. **b** Gel filtration chromatography analysis of the interaction of METTL1 (K40D/K143D/K151D/K172D) and WDR4. The left panel lists the profiles of samples applied to gel filtration, the right panel is the corresponding eluted proteins identified by SDS-PAGE.

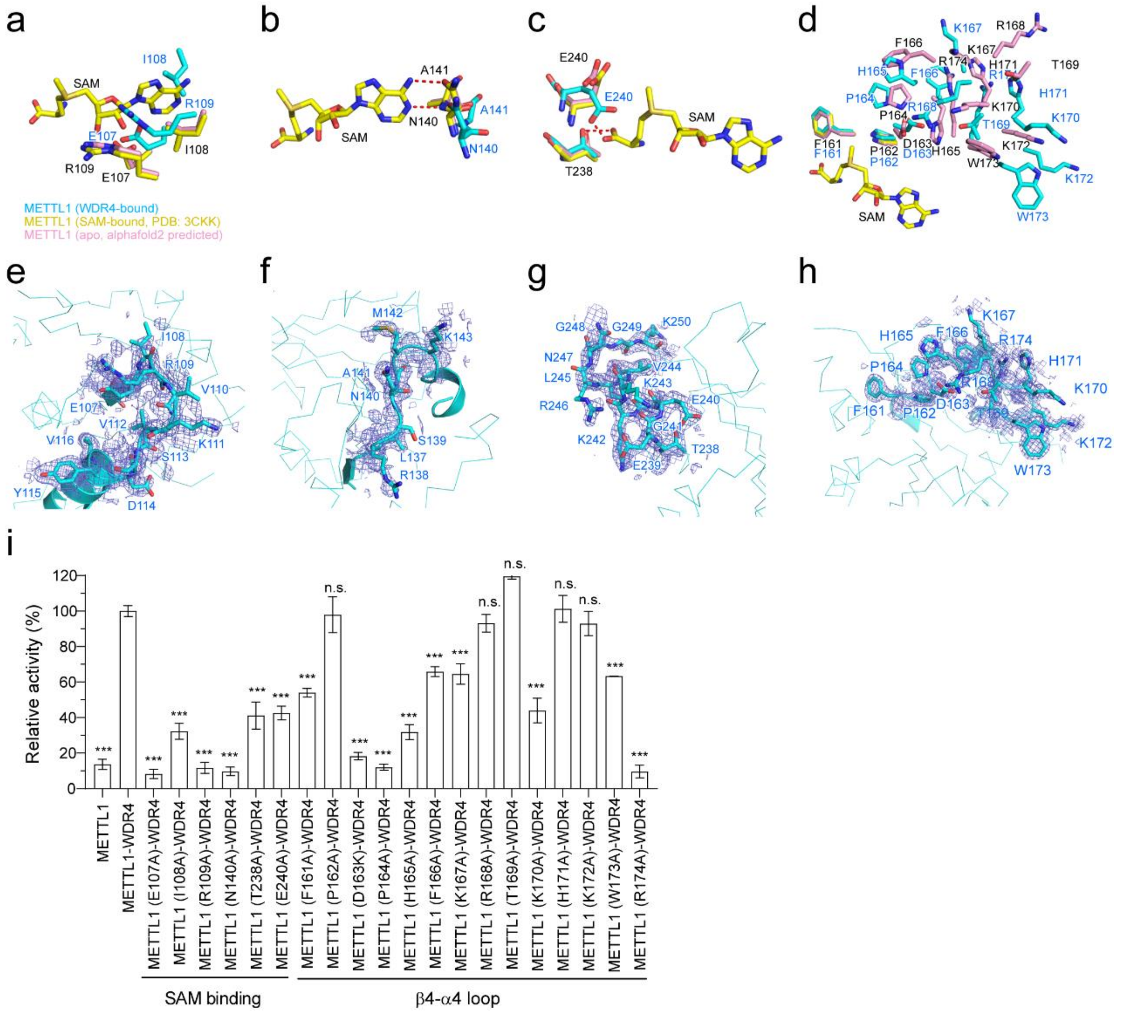

Supplementary Fig. S9 Conformational changes of METTL1 upon WDR4 binding. **a-d** Schematic representation of conformational changed regions, in which key residues are indicated by sticks. The colors are the same as in Figure 1f. **e-h** Density map (mesh) of the conformational changed regions related to **a-d**. **i** Relative methyltransferase activity of mutations. The data are the mean ± SD. Statistical analysis used two-tailed Student's t test for differences from the METTL1-WDR4 complex: ***$p < 0.001$; n.s., not significant.

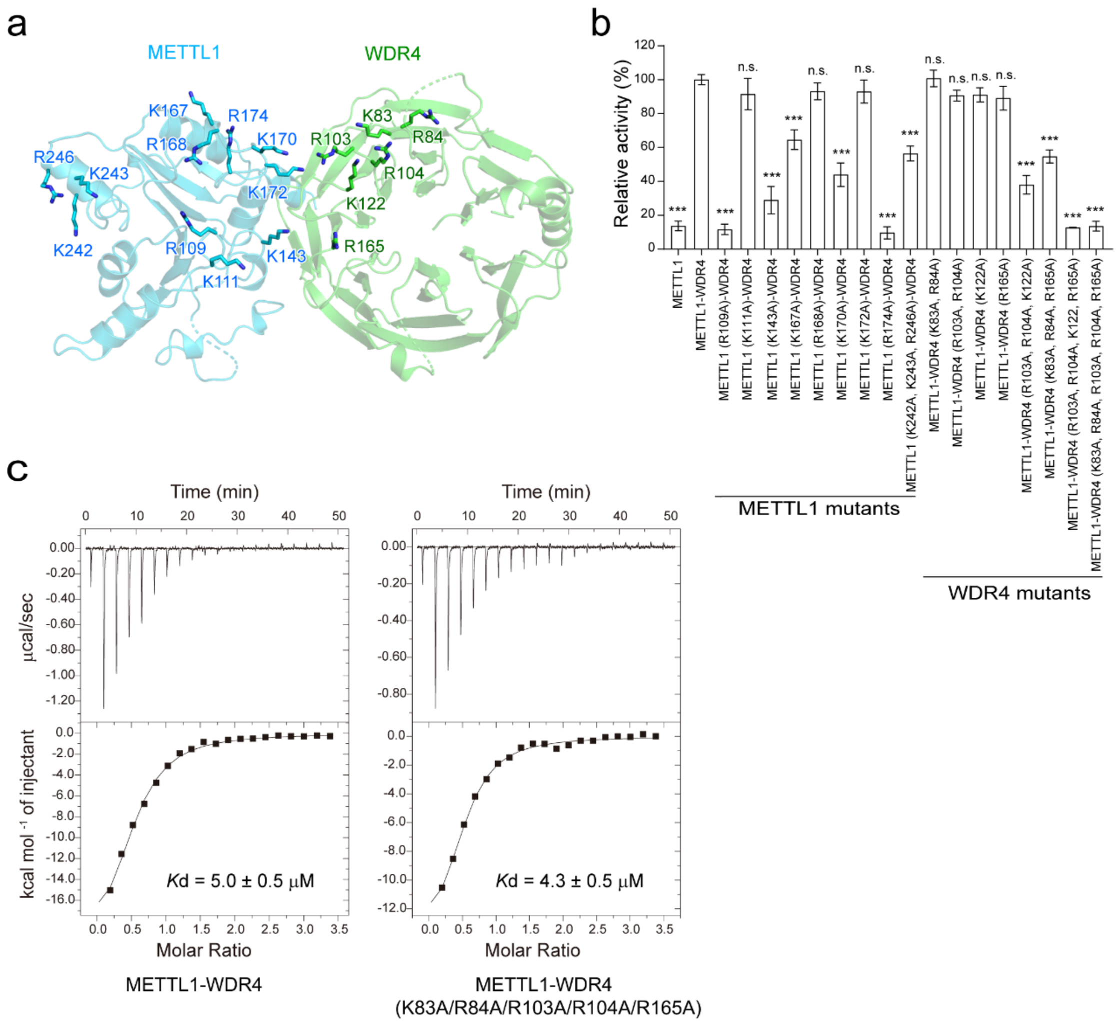

Supplementary Fig. S10 Potential tRNA-binding surface of METTL1-WDR4 complex. a Potential tRNA-binding residues in METTL1 (cyan) and WDR4 (green) are highlight in sticks. **b**, Relative methyltransferase activity of selected potential tRNA-binding site mutations. The data are the mean ± SD. Statistical analysis used two-tailed Student's t test for differences from the METTL1-WDR4 complex: ***p < 0.001; n.s., not significant. **c**, Measurement of the binding affinity between SAM and the METTL1-WDR4 complex mutations by ITC.
